# Outbreak of Peste des Petits Ruminants among Critically Endangered Mongolian Saiga and Other Wild Ungulates, Mongolia, 2016–2017

**DOI:** 10.3201/eid2601.181998

**Published:** 2020-01

**Authors:** Mathieu Pruvot, Amanda E. Fine, Charlotte Hollinger, Samantha Strindberg, Batchuluun Damdinjav, Bayarbaatar Buuveibaatar, Buyanaa Chimeddorj, Gantulga Bayandonoi, Bodisaikhan Khishgee, Batkhuyag Sandag, Jamiyankhuu Narmandakh, Tserenjav Jargalsaikhan, Batzorig Bataa, Denise McAloose, Munkhduuren Shatar, Ganzorig Basan, Mana Mahapatra, Muni Selvaraj, Satya Parida, Felix Njeumi, Richard Kock, Enkhtuvshin Shiilegdamba

**Affiliations:** Wildlife Conservation Society, Bronx, New York, USA (M. Pruvot, A.E. Fine, C. Hollinger, S. Strindberg, D. McAloose);; Wildlife Conservation Society Mongolia Program, Ulaanbaatar, Mongolia (B. Damdinjav, B. Buuveibaatar, E. Shiilegdamba);; World Wide Fund for Nature Mongolia Program, Ulaanbaatar (B. Chimeddorj, G. Bayandonoi);; Ministry of Food, Agriculture and Light Industry, Ulaanbaatar (B. Khishgee, B. Sandag);; Ministry of Nature, Environment and Tourism, Ulaanbaatar (J. Narmandakh);; State Central Veterinary Laboratory, Ulaanbaatar (T. Jargalsaikhan, B. Bataa, M. Shatar, G. Basan);; The Pirbright Institute, Surrey, UK (M. Mahapatra, M. Selvaraj, S. Parida);; Food and Agriculture Organization of the United Nations, Rome, Italy (F. Njeumi);; Royal Veterinary College, London, UK (R. Kock)

**Keywords:** Saiga tatarica mongolica, outbreak, wildlife–livestock interface, small ruminant morbillivirus, spillover, conservation impact, wild ungulates, Mongolian saiga, Mongolia, peste des petits ruminants, peste des petits ruminants virus, PPRV, viruses

## Abstract

The 2016–2017 introduction of peste des petits ruminants virus (PPRV) into livestock in Mongolia was followed by mass mortality of the critically endangered Mongolian saiga antelope and other rare wild ungulates. To assess the nature and population effects of this outbreak among wild ungulates, we collected clinical, histopathologic, epidemiologic, and ecological evidence. Molecular characterization confirmed that the causative agent was PPRV lineage IV. The spatiotemporal patterns of cases among wildlife were similar to those among livestock affected by the PPRV outbreak, suggesting spillover of virus from livestock at multiple locations and time points and subsequent spread among wild ungulates. Estimates of saiga abundance suggested a population decline of 80%, raising substantial concerns for the species’ survival. Consideration of the entire ungulate community (wild and domestic) is essential for elucidating the epidemiology of PPRV in Mongolia, addressing the threats to wild ungulate conservation, and achieving global PPRV eradication.

Peste des petits ruminants virus (PPRV; family *Paramyxoviridae*, genus *Morbillivirus*) causes an acute and highly contagious infection in domestic sheep and goats (*1*) and multiple species of wild ungulates (*2*). The resultant clinical disease, peste des petits ruminants (PPR), can lead to high morbidity and mortality rates (*3*) and is recognized as an economically important transboundary disease (*4*). The substantial effect of PPR on household-level livelihoods, well-being, food security, rural communities, and national economies have made PPR a priority for eradication (*5*–*8*). PPR is reported to affect several species of free-ranging or captive wild ruminants (*2*,*9*,*10*) but is rarely expressed clinically in wildlife populations (*11*), which were therefore thought to play a negligible epidemiologic role (*6*,*10*). Nonetheless, losses among several threatened species of wild mountain ungulates (*12*–*14*) and susceptibility of many other captive endangered ungulates (*15*–*17*) make this virus a major threat to wild ungulate conservation (*2*,*9*,*18*,*19*).

In the fall of 2016, an outbreak of PPRV among domestic sheep and goats in western Mongolia was confirmed, probably originating from uncontrolled transboundary livestock movements (*20*,*21*). In total, 83,889 small ruminants from 1,081 households were reportedly affected by PPR in 14 soums (districts) of 3 aimags (provinces), of which 12,976 small ruminants died (overall case-fatality risk 15.5%) (*22*). After this initial outbreak, control measures included vaccination of 4,632,200 sheep and 5,800,318 goats in and around the outbreak area in October 2016. Although the vaccination campaign successfully curbed the epidemic in livestock, on December 27, 2016, deaths among the Mongolian saiga antelope (subspecies *Saiga tatarica mongolica*) from PPRV infection were confirmed; later, deaths from PPRV infection of Siberian ibex (*Capra sibirica*) and goitered gazelle (*Gazella subgutturosa*) were also confirmed (*22*). In the following months, thousands of critically endangered Mongolian saiga died.

The Mongolian saiga antelope (hereafter saiga) is a nomadic antelope that now occupies <20% of its historic range in 2 provinces of Mongolia (Khovd and Gobi-Altai), representing 36,000 km^2^ of desert steppe bordered by high mountain ranges, lakes, and sand dunes (*23*). The saiga range partially overlaps that of mountain ungulates, including Siberian ibex, Argali sheep (*Ovis ammon*), and other plains ungulates such as goitered gazelle and Mongolian gazelle (*Procapra gutturosa*). It is also dominated by livestock; >1.5 million sheep and goats in the 8 soums overlapping the saiga range (*24*) are seasonally grazed over both mountain and desert steppe areas (*25*).

To describe the PPRV epizootic in the wild ungulate community of Mongolia, we gathered all available evidence from field missions, histopathology examinations, government records, wildlife population monitoring efforts, and laboratory testing (including molecular characterization of the causative agent). We describe the significance of our findings for the global PPR eradication program and the conservation of wild ungulate species (Figure 1).

**Figure 1:**
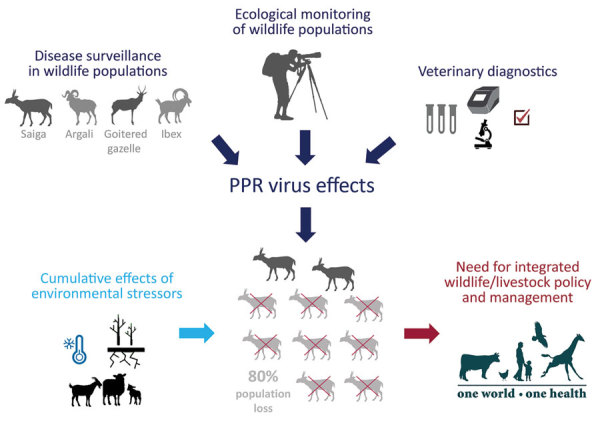
Summary of study of the 2016–2017 outbreak of peste des petits ruminants among wildlife, Mongolia. PPR, peste des petits ruminants.

## Materials and Methods

### Emergency Field Mission

On January 20, 2017, shortly after the first confirmation of PPRV infection in saiga, the Crisis Management Center–Animal Health, led by the Food and Agriculture Organization of the United Nations and the World Organisation for Animal Health, and the Mongolia government organized an investigation of PPR among wild and domestic animals (*22*). Reports of cases before the first official confirmation were collected from multiple sources in the field (Appendix section 1.1). In addition, samples were obtained from 27 animals that were found sick (2 saiga) or dead (2 ibex, 2 goitered gazelle, 21 saiga) during direct field observations. For dead animals, full necropsy examinations were performed when possible (2 ibex, 2 goitered gazelle, 9 saiga); necropsy reports were available for 2 ibex, 1 goitered gazelle, and 4 saiga. Heads were collected from the rest of the animals (12 saiga). Tissues were available for histologic examination from 17 animals (2 ibex, 2 goitered gazelle, 13 saiga) (Appendix section 1.2). We used the lateral flow device Peste-Test (The Pirbright Institute, https://www.pirbright.ac.uk) as a rapid penside field test for PPRV (*26*) on eye swab samples from 20 animals (2 ibex, 1 goitered gazelle, 17 saiga), except for 1 live saiga from which we noninvasively obtained feces and saliva. We sent samples to the State Central Veterinary Laboratory (SCVL) for PPRV confirmation by use of gel-based reverse transcription PCR (RT-PCR) (Appendix section 1.3) (*27*). For 2 of the saiga samples, the Pirbright Institute sequenced the C-terminus portion of the N-gene (Appendix section 1.4).

### Phylogenetic Analysis

We retrieved and used the partial N-gene sequences of PPRV (n = 56) available in GenBank for southern, central, and eastern Asia through September 2018 for constructing a neighborhood-joining phylogenetic tree. Sequences included the 2 partial N-gene sequences obtained from saiga from Mongolia in this study (Appendix section 1.4).

### Reporting to SCVL

All wildlife samples submitted to SCVL in 2016–2017 were compiled in a dataset, which included georeferences and PPR diagnostic results when available. Most samples were tested by using the RT-PCR procedure mentioned above, and a subset of samples was also tested by using ID Screen PPR Competition and ID Screen PPR Antigen Capture (IDvet, https://www.id-vet.com). Cases were considered positive when >1 of the 3 test results was positive. When georeferences were missing, we used the location description to determine the approximate geographic coordinates and mapped it by using ArcGIS 10.2 (ESRI, https://www.esri.com). These coordinates were used to trace the spatio-temporal progression of the PPR outbreak in wildlife, including identifying potentially undiagnosed wildlife illness and deaths that may have been part of the same outbreak.

### Government Carcass Disposal

From January 8 through February 28, 2017, as part of the Mongolia government emergency response (Appendix section 2) in the Khovd and Gobi-Altai Provinces, livestock movements were restricted and saiga carcasses were collected and destroyed. Carcass disposal was conducted at 8 sites where records were kept of the total number of carcasses and sex of the animal (when available). In some soums, at the initiative of the soum-level government, the count and collection of carcasses was maintained until June 30, 2017.

### Saiga Population Surveys

In 2010, distance sampling (*28*) was first applied to the saiga population as a way to improve population abundance estimates (*23*). Thereafter, it was implemented as part of a routine monitoring program; local saiga rangers conducted distance sampling surveys along 40 transects ranging from 2 to 99 km, for a total of 1,505 km of survey effort. Each survey was conducted by 4 trained teams, who drove vehicles along transects and recorded for each group of saiga seen the radial distance, angle from the transect line, and group size. To better monitor the population-level effect of the outbreak, we repeated the surveys in January, March, and May 2017, and April 2018. 

Following systematic data cleaning steps (Appendix section 3.1; datasets, ; R code available upon request), we used Distance 7.2 software (*29*) to fit detection function models to the distance sampling data. Models were fitted separately for each survey and, when sample size was sufficient, were stratified by the 3 regions within the home range (Durgun Steppe, Khuisiin Gobi, and Sharga Gobi). In addition, to estimate population density and abundance, we assessed group size bias (e.g., when smaller groups farther from the transect line tend to be missed) and corrected when necessary (Appendix sections 3.2).

### Ethics Considerations

No ethics approval was required for the outbreak response because the investigation was a response to an emergency situation, and no live animal handling was required to obtain the samples (samples obtained from dead animals or from environmental recovery of excreted/secreted material). The driving transect survey technique for estimating the population of Mongolian saiga was reviewed by the wildlife research advisory committee of the Mongolian Academy of Sciences. Members of the Mongolian Academy of Sciences act as the main scientific advisors to the Ministry of Environment in issuing of permits related to wildlife research in Mongolia.

## Results

### Clinical Manifestations of PPRV Infection in Wild Ungulates

**Figure 2:**
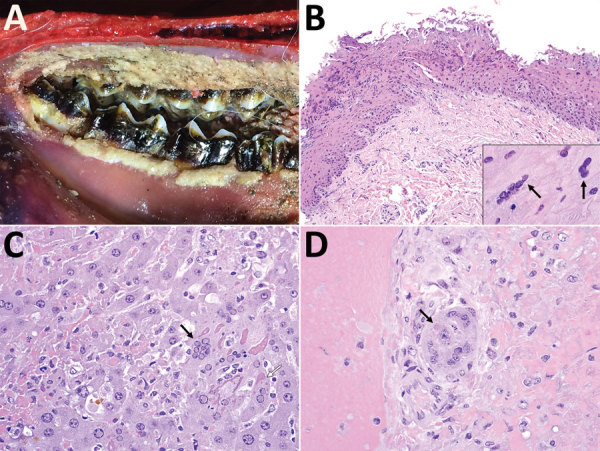
Macroscopic and microscopic lesions of peste des petits ruminants virus–infected saiga, Mongolia, 2016–2017. A) Erosion and necrosis of the oral mucosa along the gingival margin of the molar teeth. B) Erosion and necrosis of the superficial oral mucosa with multifocal epithelial syncytia (inset, arrows). Original magnification ×200; inset ×1,000. C) Multifocal hepatocellular necrosis (upper and lower left, upper right) with dissolution of hepatic cords, occasional hepatocellular syncytia (black arrow), and prominent eosinophilic viral inclusion bodies, both intranuclear and chromatin (black arrow) and globular to amorphous within the cytoplasm (white arrow). Original magnification ×400. D) Bile ductule showing eosinophilic intraepithelial intracytoplasmic viral inclusion (arrow) and mild cellular degeneration with focal luminal cellular debris. Original magnification ×600.

One live clinically ill saiga (confirmed positive for PPRV by Peste-Test) could be approached and displayed the following clinical signs: lethargy with tachypnea and dyspnea, seropurulent ocular discharge with staining of the suborbital area, salivation, diarrhea, and weakness. Of the 20 animals tested with Peste-Test, results were positive for 17 (1 ibex, 1 goitered gazelle, and 15 saiga); of the 17 animals tested by RT-PCR, results were positive for 16. Of the 22 total tested animals, 20 were positive by either Peste-Test or RT-PCR. For the 6 animals PPRV positive by RT-PCR that underwent necropsy (4 saiga, 1 goitered gazelle, 1 ibex), notable gross pathology findings included emaciation (n = 4), red nasal mucosa or discharge (n = 3), erosive to ulcerative lesions of the oral mucosa (n = 3), red or consolidated portions of lungs (n = 6), and red discoloration of the intestinal mucosa or presumptive enteritis (n = 4). Tissues available for histology from PPRV-infected animals (positive test result, histologic evidence, or both) showed acute cellular degeneration and necrosis, with varying degrees of associated acute inflammation, that affected the oral/pharyngeal mucosa, hepatocytes, cholangiolar epithelium of bile ductules, bronchiolar epithelium, and intestinal crypt epithelium (Table 1; Figure 2). We observed viral inclusion bodies and a few viral syncytia to varying degrees in oral/pharyngeal, liver, lung, and intestinal lesions. In some cases, postmortem artifacts hindered intestinal evaluation. Concurrent diseases in PPR-infected animals were found in 1 saiga with stomatitis typical of parapoxviral infection (contagious ecthyma) and 1 goitered gazelle with bacterial sepsis. Atrophy of adipose tissue and lymphoid depletion were identified in animals with and without evidence of PPRV infection (Appendix section 1.2; individual animal data, https://doi.org/10.6084/m9.figshare.7502258.v1).

**Table 1:** Major histologic lesions in animals infected with peste des petits ruminants and concurrent diseases, Mongolia, 2016–2017*

Lesion or disease	Animal ID nos.											
	Mongolian saiga								Goitered gazelle			Ibex
	4	5, 8†	9	10	11	17	13, 15†		18	25		20
PPRV-specific lesions												
Oro/pharyngeal mucosa: erosion, epithelial necrosis, multifocal, acute (stomatitis, necrotizing)	+++, S+	+++, S+	+, S+	–	–	+++, S+, I+ (IN)	–		+, S+	NE		NE
Liver: degeneration and necrosis, hepatocytes, multifocal, random, acute (hepatitis, necrotizing)	NE	NE	++, S+, I+++ (IC and IN)	++	+	++	NE		++, S+, I+ (IN)	++, S+, I+ (IN>IC)		NE
Liver: degeneration and necrosis, biliary epithelium, bile ductules, multifocal, acute	NE	NE	+, I++ (IC>IN)	–	–	–	NE		+	++ I+ (IN>IC)		NE
Liver: cholestasis, canalicular, acute	NE	NE	+	+	–	–	NE		–	–		NE
Liver: hyperplasia, bile ductules, chronic	NE	NE	+	++	–	–	NE		–	–		NE
Lung: degeneration and necrosis, bronchiolar epithelium, multifocal, acute	NE	NE	+, S+, I+(IN and IC)	–	–	–	NE		–	++ S+ I++ (IN and IC)		NE
Intestine: necrosis, crypt epithelium, multifocal, acute	NE	NE	+, I+ (IC>IN)	–/PMA	–	–/PMA	NE		–/PMA	–/PMA		NE
Concurrent diseases	NA	NA	NA	NA	Proliferative stomatitis, parapoxvirus suspected	NA	NA		NA	Bacterial sepsis with bacteremia		NA

*I, presence of viral inclusion bodies; IC, intracytoplasmic inclusion; ID, identification; IN, intranuclear inclusion; NA, not applicable; NE, not examined; PMA, assessment hindered by postmortem autolysis; S, presence of syncytia; +, mildly severe; ++, moderately severe; +++, severe; –, lesion not found. †Tissue samples pooled.

### Phylogenetic Analysis

**Figure 3:**
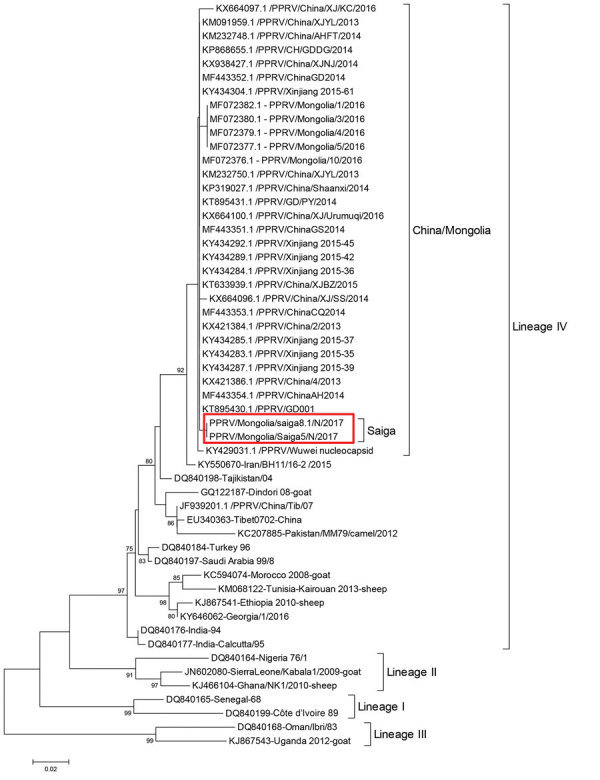
Neighbor-joining tree constructed on the basis of partial N-gene sequences of peste des petits ruminants virus (PPRV), showing relationships among the PPRV isolates. The Kimura 2-parameter model was used to calculate percentages (indicated by numbers beside branches) of replicate trees in which the associated taxa clustered together in 1,000 bootstrap replicates. Red rectangle outlines the 2 PPRV sequences from saiga obtained from this study (BankIt2279588 MOG/saiga5-2017, GenBank accession no. MN648447; BankIt2279588 MOG/saiga8.1-2017, accession no. MN648448). GenBank numbers are indicated. Scale bar indicates nucleotide substitutions per site.

We obtained partial N-gene sequences from 2 PPRV-infected saiga. The phylogenetic analysis, conducted by using 58 partial N-gene sequences (Figure 3), confirmed that the PPRV sequences were of PPRV lineage IV and formed 1 cluster with sequences from livestock in Mongolia in 2016 (*20*) and from outbreaks in China in 2013–2016 (Figure 3). In addition, these sequences are genetically close to sequences from central Asia (i.e., Iran [GenBank accession no. KY550670] and Tajikistan [accession no. DQ840198]).

### Mapping of Confirmed and Unconfirmed Cases

**Figure 4:**
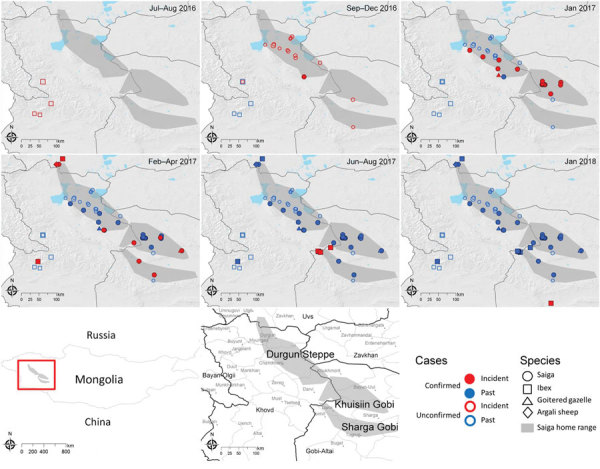
Spatiotemporal distribution of confirmed and unconfirmed cases of peste des petits ruminants (PPR) in several wild ungulate species in Mongolia. Each panel illustrates cases that occurred during the panel-specific time period (incident cases) and cases that occurred during previous periods (past cases). In the 2 periods before the first laboratory confirmation of PPR in saiga in December 2016, 2 clusters of unconfirmed cases (open shapes) were documented and matched the pattern of livestock PPR case distribution. In January 2017, the outbreak spread rapidly through the saiga population and expanded southward and northward from February 2017 through May 2017, when the last confirmed saiga cases were reported. Subsequent PPR cases involved ibex until at least January 2018. Maps at bottom show location of study area in Mongolia and specific location names.

From the onset of the outbreak in livestock to December 2018, samples from 30 georeferenced individual animals of 4 species (23 saiga, 5 ibex, 1 argali sheep, and 1 goitered gazelle) were submitted to and confirmed PPRV positive by the SCVL (Figure 4; case mapping data, ). Most of the early cases in saiga (until January 2017) were detected in the Durgun Steppe and the Khuisiin Gobi portions of the saiga home range (particularly in Chandmani Soum, Khovd Province); the cases in the Sharga Gobi portion of the saiga range and the rest of the Gobi-Altai Province first appeared in February 2017 (Figure 4). After the last reported saiga case in May 2017, all subsequent confirmed cases were in ibex that died in the Gobi-Altai Province through January 2018.

Suspected but unconfirmed cases were documented through interviews conducted during the Crisis Management Center–Animal Health field mission, review of provincial and central laboratory records of undiagnosed wildlife deaths, and interviews with local herders and rangers patrolling the saiga range (Figure 4). Unconfirmed cases revealed 2 clusters, 1 in the southwestern part of Khovd Province close to the border with China and 1 between Khar-Us and Durgun Lake north of the saiga range. The first cluster involved 10 ibex with severe diarrhea that led to death at 3 locations in July and August 2016. At that time, PPR was not suspected because the outbreak in livestock was not confirmed and declared until September 2016 (although retrospective serologic evidence indicates that PPRV was probably circulating in livestock as early as November 2015 [*22*]). The second cluster was reported by herders, who indicated substantial saiga deaths in December 2016, before the first PPR diagnosis in saiga was confirmed on December 27, 2016. Saiga rangers confirmed the death of at least 27 animals in these locations. This pattern mirrors quite closely the PPR outbreak observed in livestock; initial cases in livestock clustered at the southwestern Mongolia–China border and at a secondary outbreak focus in the Khar-Us Lake area (*22*).

### Government Carcass Disposal

From January through February 2017, the emergency response team collected and destroyed 4,202 saiga carcasses. Adding the soums for which collection continued until June 2017, the collection efforts totaled 5,425 saiga, 41 goitered gazelles, and 24 ibex. Among soums for which information on animal sex was recorded (Bayan-Uul, Darvi, Khukhmorit and Sharga), sex ratios ranged from 2 to 6 females for 1 male. The absence of information on exact carcass locations, search routes, and search efforts prevented further assessment of the comprehensiveness of carcass collection and of the spatial distribution of the carcasses.

### Saiga Population Surveys

**Figure 5:**
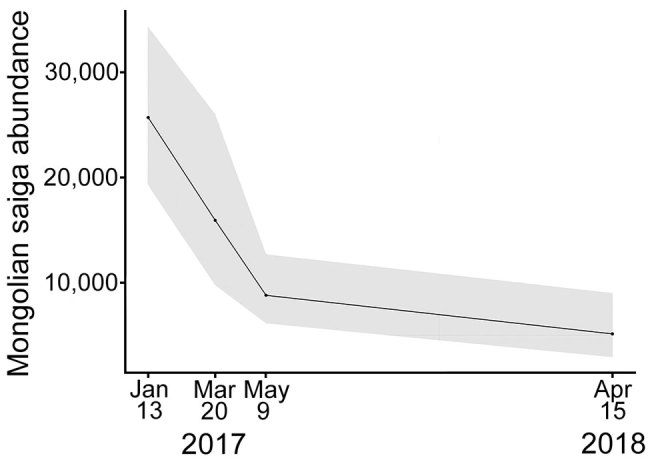
Saiga population in Mongolia during the outbreak of peste des petits ruminants in 2017 and the next year. Shaded area represent 95% CIs around abundance estimates.

Surveys conducted in January, March, and May 2017 and April 2018 indicated a steep decline in direct saiga observations along transects, from 2,130 saiga in 328 groups in January 2017 to 369 saiga in 46 groups in April 2018, despite similar survey efforts (Table 2). Abundance estimates provided by the best model for each period (Appendix section 3.3) confirmed the saiga population decline from 25,699 (95% CI 19,249–34,310) in January 2017 to 8,806 (95% CI 6,095–12,721) by May 2017, the last month of reported saiga deaths. However, the saiga population continued declining after May 2017; the last survey in April 2018, almost a year after the outbreak, showed an estimated abundance of 5,142 (95% CI 2,929–9,028), 20% of the January 2017 population size (Figure 5). The average probability of detecting live animals in the surveyed area ranged from 0.38 to 0.59.

**Table 2:** Summary statistics from saiga distance sampling surveys conducted in study of outbreak of peste des petits ruminants among critically endangered wild ungulates, Mongolia, January 2017–April 2018*

Date	Days since first PPR confirmation	Total effort, km	No. individuals (groups)	Density of individuals (95% CI)	Abundance (95% CI)	Expected cluster size (95% CI)	Average probability of detecting live saiga
2017 Jan	30	1,505	2,130 (321)	1.18 (0.88–1.58)	25,699 (19,249–34,310)	D: 3.9 (3.2–4.8); K: 4.1 (3.4–4.9); S: 6.3 (4.8–8.4)	D: 0.35 (0.29–0.43); K: 0.37 (0.31–0.43); S: 0.42 (0.35–0.50)
2017 Mar	90	1,505	1,999 (148)	0.73 (0.45–1.20)	15,933 (9,759–26,011)	D: 10.9 (7.5–16.1); K: 13.5 (9.5–19.2); S: 5.4 (4.2–7.0)	D: 0.44 (0.36–0.55); K: 0.69 (0.51–0.94); S: 0.60 (0.50–0.71)
2017 May	150	1,263	742 (157)	0.41 (0.28–0.58)	8,806 (6,095–12,721)	D: 2.6 (2.1–3.3); K: 2.7 (2.2–3.3); S: 2.4 (1.7–3.6)	0.51 (0.45–0.57)
2018 Apr	480	1,505	369 (46)	0.24 (0.14–0.42)	5,142 (2,929–9,028)	D: 11.7 (7.5–18.1); K: 10.9 (6.1–19.6); S: 6.3 (4.4–8.9)	0.51 (0.40–0.65)

*D, Durgun Steppe; K, Khuisiin Gobi; PPR, peste des petits ruminants; S, Sharga Gobi.

## Discussion

Epidemiologic, pathology, and ancillary test findings in this PPR outbreak in Mongolia support the diagnosis of PPRV infection causing wild ungulate deaths across both desert steppe and mountain ecosystems. Saiga antelope were confirmed to be susceptible and capable of spreading PPRV infection within their population over a short time, suggesting high viral excretion loads and contact rates over the period of the epidemic. PPR in 3 other species of antelope in a semicaptive private collection in the United Arab Emirates has been previously reported (*30*), and our report indicates that free-ranging antelope exhibit the same level of susceptibility. The multiple clusters of ibex deaths suggest multiple spillovers from livestock followed by effective intraspecies transmission. The occurrence of apparently sporadic deaths among goitered gazelle and Argali sheep confirms the susceptibility of these species but raises questions about their ability to spread infection.

The pathologic lesions found in this outbreak were largely consistent with typically reported PPR lesions in domestic species (*31*) (Appendix section 1.2). The prominence of liver lesions and involvement of biliary epithelium were unusual, although they have been reported for other morbillivirus infections, including infection of wild lesser kudu (*Tragelaphus imberbis*) by the closely related rinderpest virus (*32*). Pathologic findings in the examined animals were not consistent with hemorrhagic septicemia caused by *Pasteurella multocida,* which caused large-scale saiga mortality in Kazakhstan (*33*,*34*). Diminished fat reserves and lymphoid depletion in PPR-positive and PPR-negative saiga was a nonspecific finding and could indicate the existence of environmental stressors, possibly explained by midwinter conditions. Further research is required to assess whether poor body condition and potential immunosuppression could contribute to virulent expression of PPRV in these animals. The presence of at least 2 pathogens of livestock in the examined saiga also suggested a high permeability of this livestock–wildlife interface to infectious diseases, which may have contributed to the overall mortality rate.

The 2 PPRV N-gene sequences obtained from infected saiga were similar to sequences obtained from livestock during outbreaks in Mongolia in 2016 and China in 2013–2016. This similarity is consistent with a spillover of PPRV from infected domesticated sheep and goats to wild ungulates. In addition, these sequences were genetically close to sequences from central Asia (i.e., Iran [GenBank accession no. KY550670] and Tajikistan [accession no. DQ840198]) but different from sequences from China in 2007 (accession nos. EU340363 and JF939201) (Figure 3), which suggests recent spread of PPRV from central Asia to China and then to livestock and wild ungulates in Mongolia.

PPRV outbreak mapping suggested that wildlife may have been infected earlier (possibly July 2016) than the first confirmed case (December 2016) and that wildlife infections closely followed the timing of the livestock outbreak. The absence of laboratory confirmation of PPRV infection for these initially unconfirmed clusters warrants cautious interpretation, but strong epidemiologic evidence indicates that these suspected cases were part of the same PPRV outbreak. The apparent spatial discontinuity between the 2 outbreak foci supports the hypothesis that the spread of PPR was mainly driven by livestock movement, because the wild mountain ungulates (ibex in the first putative outbreak focus) are relatively resident and unlikely to move long distances across multiple ecotypes. This spatial discontinuity also suggests multiple spillover events from livestock to different wildlife populations, which will require further analysis based on genetic data. 

The early onset of PPRV in ibex and the lower and more prolonged incidence of cases in this species (at least until January 2018) are in contrast with the rapid transmission through the saiga population (apparently ceased by June 2017). This contrast in incidence suggests different dynamics of PPRV transmission in the 2 species, influenced by population structure, habitat, and interspecies–intraspecies interactions. Further work, including identification of shared resources between species (e.g., watering points, residual snow patches, and mineral licks), contact rates, and modeling should be conducted to better determine the most likely transmission routes and the respective roles of these wild and domestic ungulates in this multihost system. The probable 5-month delay between the first unconfirmed cases documented and the first confirmation in saiga underscores the value of maintaining operational wildlife health surveillance systems for early detection of wildlife illness and deaths.

The initial mortality estimates, obtained from carcass collection and disposal efforts, were probably underestimated because of imperfect detection (*35*–*38*) (Appendix section 3.3). If systematic carcass removal is determined to be cost-effective, adopting standard ecological monitoring methods to ensure reliable and unbiased characterization of mortality patterns is imperative (*39*). The most compelling estimate of the population-level effect of the PPRV outbreak in saiga was, therefore, derived from the population monitoring efforts by using distance sampling methods (which account for imperfect detection), indicating a saiga population decline of >80%. These estimates depict a serious situation for the Mongolian saiga population and a substantial setback after >10 years of conservation efforts to secure saiga population recovery after a historical low in the early 2000s (*40*,*41*). The significance of this event to saiga goes beyond the Mongolian subspecies because other unrelated mass mortality events have recently affected the species and are threatening its global conservation (*33*). Although saiga have shown great potential for recovery (*42*), in part because of fertility and frequently giving birth to twins or triplets (*43*,*44*), the population estimates a year after the outbreak showed little evidence of recovery. The timing of the outbreak just after rut season (which may have facilitated transmission because of congregation of animals) and during gestation probably delayed recovery through effects on recruitment. In addition, very cold temperatures with exceptionally heavy snowfall (known as dzud) also resulted in saiga deaths during winter 2018 and probably contributed to the additional population decline from June 2017 through April 2018 (Figure 5). We cannot exclude as potential causes for the sustained population decline the cumulative effects of multiple factors, other concurrent conditions, and undetected PPRV circulation. The lack of similarly detailed data for the other species of ungulates prevented assessment of the full conservation effect of the outbreak, but deaths across the ungulate community suggest broader effects on these ecosystems.

Factors that favored the eradication of rinderpest included an expectation that wildlife did not act as a reservoir of infection for domestic animals (*8*). This multispecies mass mortality event in Mongolia and recent similar events in eastern Asia and the Middle East (*18*) challenge the assumption that wildlife play a negligible role in the epidemiology and ecology of PPRV. This observation has substantial implications for the current global eradication program and efforts to outline National Strategic Plans for PPR control and eradication. The explicit integration of wildlife protection into these National Strategic Plans should be considered, and plans should include setting livestock vaccination targets that can effectively prevent spillover of virus from livestock to other susceptible wildlife (*45*).

The growing number of livestock on rangelands of low productivity, such as in Mongolia and much of central Asia (*26*,*46*,*47*), exerts increasing pressure on sympatric wild ungulates through competition for resources (*48*,*49*). Restricted access of wild ungulates to quality forage, water, and minerals may result in poor nutritional status and immune function (*50*), possibly reducing their resilience to livestock pathogens to which they are increasingly exposed. Global changes in climate and expected shifts in species distributions and habitat suitability may further reduce resource availability and increase wildlife–livestock interactions. Evidence for possible dislocation of species-habitat-climate relationships leading to increased susceptibility to disease can be found in the mass mortality that occurred because of hemorrhagic septicemia in another subspecies of saiga (*Saiga tartarica tartarica*) in Kazakhstan (*34*). These combined factors could result in an increasing number of disease spillover events, followed by rapid amplification in populations already under multifactorial stresses. To ensure that objectives of rural development and biodiversity protection are compatible and jointly met, integration of livestock and wildlife management must be improved (Figure 1). Doing so proactively in the face of global climate change and increasing demands of a growing global population is a critical challenge of this century.

Appendix Supplemental methods and results for study of outbreak of peste des petits ruminants among critically endangered wild ungulates, Mongolia, 2016–2017.
