# Supplementary material for: Outbreak of Peste des Petits Ruminants among Critically Endangered Mongolian Saiga and Other Wild Ungulates, Mongolia, 2016–2017
Source: Emerg Infect Dis. 2020 Jan;26(1):51–62. doi: 10.3201/eid2601.181998 (PMC6924898; doi:10.3201/eid2601.181998)
Supplement: Appendix — Supplemental methods and results for study of outbreak of peste des petits ruminants among critically endangered wild ungulates, Mongolia, 2016–2017. [file 18-1998-Techapp-s1.pdf]

Article DOI: https://doi.org/10.3201/eid2601.181998

Outbreak of Peste des Petits Ruminants among Critically Endangered Mongolian Saiga and Other Wild Ungulates, Mongolia, 2016–2017

Appendix

1. Emergency field mission

1.1 Interviews during field mission

During the field mission organized by Food and Agriculture Organization of the United Nations (FAO)/ World Organisation for Animal Health (OIE) / Crisis Management Center-Animal Health (CMC-AH) and the Mongolian government, meetings were held with local officials, veterinarians, herders, and local offices of international NGOs [Wildlife Conservation Society (WCS) and World Wide Fund for Nature (WWF)]. Reports of cases before the first official confirmation were collected from these sources.

1.2 Pathology

A rapid response to the wildlife mortality enabled preliminary pathologic description of PPR in saiga, goitered gazelle and ibex. Tissues were fixed in 10% neutral phosphate-buffered formalin for at least 24 hours and processed routinely for histologic examination. The number of organ tissues available for histology (from 13 saiga, 2 ibex, 2 goitered gazelle) varied from animal to animal based on ability to conduct a full or partial necropsy under field conditions. Tissues were pooled for two pairs of saiga and each pair was counted as one case in the histological result section. Some tissue types (e.g., oral/pharyngeal mucosa, lymph nodes) commonly included multiple samples per case, but were not specifically anatomically differentiated.

Gross necropsy observations were hindered by field conditions including post-mortem condition and inter-observer variation. The most significant repeated macroscopic observation

was mucosal (oral or nasal) reddening and erosion, which was corroborated by mucosal lesions identified in all of these cases histologically, though not all PPRV+ cases had histologically identified mucosal lesions in the available samples. The macroscopic observation of intestinal reddening correlated with necrosis in one of the cases (saiga) in which it was observed; the others were too autolyzed for reliable intestinal assessment (1 saiga, 1 goitered gazelle), or not examined (1 ibex). The majority of cases (4/6) in which red lung discoloration was observed macroscopically correlated with inflammatory lesions typical of aspiration pneumonia. In one saiga there were concurrent (milder) lesions of PPRV. The lung discoloration seen in the remaining saiga correlated with autolysis and freezing artifact, and that seen in the ibex was not available for histologic examination. Evidence of aspiration was also identified in lung of 2/4 saiga with no evidence of PPRV infection, and was considered a common sequella of illness or debilitation, not directly linked to PPRV status. An additional gross finding was enlarged or red discolored lymph nodes in body cavities in four PPRV-positive cases (3 saiga, 1 goitered gazelle). This correlated histologically with sinus histiocytosis and/or congestion in three cases and was not examined in one saiga. However, post-mortem carcass condition and emaciation may have complicated assessment of lymph node size and color. The finding was observed in both PPRV-positive and negative animals and was not consistent across those infected.

Histologically, the oral/pharyngeal mucosa in 4/7 examined saiga and 1/1 goitered gazelle was disrupted by mild to marked multifocal erosion to ulceration with epithelial degeneration and necrosis, variable inflammation (necrotizing stomatitis), rare syncytial cells in all cases, and intranuclear viral inclusion bodies in one saiga. The degree of inflammation correlated with surface bacterial colonization. The liver demonstrated acute, multifocal, random hepatocellular degeneration and necrosis with varying absent to mild acute inflammation (necrotizing hepatitis) (4/4 saiga and 2/2 goitered gazelle), as well as intranuclear and/or intracytoplasmic viral inclusion bodies and syncytia in one saiga and both goitered gazelle. In one of the saiga and both goitered gazelle, there was additionally mild to moderate necrosis of cholangiolar epithelium, with intranuclear and intracytoplasmic viral inclusions in the saiga and one goitered gazelle. Mild cholestasis, predominantly intracanalicular, was noted in association with the liver lesions in two affected saiga. Additional viral histologic lesions included mild to moderate bronchiolar epithelial necrosis multifocally in the lung (1/4 saiga and 1/2 goitered gazelle) and mild multifocal necrosis of crypt epithelium in the intestine (1/4 saiga and 0/2

goitered gazelle), with few syncytia and inclusion bodies (intranuclear and intracytoplasmic) at both sites. Post-mortem artifacts hindered intestinal evaluation some cases. Concurrent diseases in PPR-infected animals included one saiga with stomatitis typical of parapoxviral infection (contagious ecthyma) and one goitered gazelle with bacterial sepsis as characterized by mild foci of inflammation with intralesional bacteria in the heart and liver and intravascular bacteria in the kidney. Atrophy of adipose tissue was histologically confirmed in all cases in which adipose tissue was examined (3/3 PPRV-positive and 3/3 PPRV-negative animals). Lymphoid depletion in lymph nodes and/or spleen was identified histologically in 7/8 and 3/4 PPRV-positive and –negative samples, respectively. Among the animals without evidence of PPRV (4 saiga, 1 ibex), two saiga had histologic evidence of other infections (disseminated mycosis and small intestinal endoparasitism, respectively). The detailed observations for each animal are available online https://doi.org/10.6084/m9.figshare.7502258.v1)

1.3 Laboratory diagnostic procedures

Samples sent to the State Central Veterinary Laboratory (SCVL) for PPRV confirmation were processed usinga gel-based reverse transcription polymerase chain reaction (RT-PCR), carried out according to Couacy-Hymann et al. (2002) (*1*) using Qiagen One-step RT-PCR kit (QIAGEN). Additional diagnostic procedures carried out on cases submitted to the State Central Veteriary Laboratory included antibody detection using ID Screen ® PPR Competition (IDvet, Montpellier, France), antigen detection using ID Screen ® PPR Antigen Capture (IDvet, Montpellier, France), antigen detection using Peste-Test® (The Pirbright Institute, UK).

Detailed results of these tests are available in the data file https://doi.org/10.6084/m9.figshare.7502264.v1

The combined results of histology examination and these additional diagnostic procedures provide a strong support for PPRV being the causative agent of the observed mortality.

1.4 Phylogenetic Analysis

On two of the saiga samples, sequencing of the C-terminus portion of the N-gene were performed by The Pirbright Institute, UK, following shipment of samples on dry ice. Tissue samples were homogenized and total RNA extracted following previously described methods (*2*). Viral RNA was reverse transcribed and amplified as previously described (*3*) using the

superscript III One-Step RT-PCR kit (Invitrogen, Carlsbad, USA). PCR amplicons were purified using the GE Healthcare Illustra GFXPCR purification kit (GE Healthcare, Pittsburgh, USA) according to the manufacturer's instructions and sequenced using BigDye® Terminator v3.1 Cycle Sequencing Kit (Applied Biosystems, Carlsbad, USA) on an ABI 3730 machine. Sequences were assembled and analyzed using SeqMan pro (DNAStar Lasergene 13.0).

The partial N-gene sequences of PPRV (n = 56) available in GenBank for South, Central, and East Asia through September 2018 were retrieved and used for constructing a neighborhood-joining phylogenetic tree, including the two partial N-gene sequences obtained from Mongolian saiga in this study. Alignments of the N-gene sequences were made using the Clustal W program and used for construction of distance matrices using the Kimura 2-parameter nucleotide substitution model (*4*) as implemented in the programme MEGA 6.0 (*5*). A maximum-likelihood phylogenetic tree was generated using MEGA 6.0, and the robustness of tree topology was assessed using 1,000 bootstrap replicates.

2. Government carcass disposal

Following the beginning of the outbreak in saiga, the Inter-Ministerial Working Group on Saiga Mass Mortality was created and included the Ministry of Nature Environment and Tourism (MNET), the Ministry of Food, Agriculture and Light Industry (MoFALI), the National Emergency Management Agency (NEMA), the General Agency of Specialized Inspection (GASI), SCVL, and representatives of academic institutions, non-governmental conservation organizations, and other international organizations.

This working group initiated the implementation of an emergency response together with provincial-level counterparts of these agencies in the Khovd and Gobi-Altai provinces, and proceeded to restrict livestock movements, and collect and destroy saiga carcasses, between January 8 and February 28, 2017. This effort was supported by local veterinarians, citizens, non-governmental organization staff, provincial government officials, and other international experts.

3. Saiga Population surveys

3.1 Data preparation

Despite efforts to standardize data collection, deviations from the protocol were reported by field biologists, or identified following initial data inspection. These broadly fall under three categories: 1) observations made outside of transects, 2) observations made after spending an excessive amount of time in one location along the transect, and 3) duplicate observations of the same individual or group. To address these issues in the data we excluded observations that: 1) did not align on transects, effectively resulting in an initial truncation of observations made over 2 km away from any transect line, 2) were made after 10 minutes from the same location along the transect, and 3) were less than 500 m distance from a group of the same size (+/–4 individuals) based on exact group location (calculated using the bearing and distance from the point of observation; R code available upon request for procedure 2 and 3).

The May 2017 survey was particularly problematic, with significant missing data due to a gap in scientific supervision of field staff. These missing data seemed to cluster on particular transects, and when this was the case, the entire transect was excluded from the data (thereby reducing the overall survey effort for this time point).

3.2 Data analysis

Distance sampling data were analyzed using the software Distance v7.2 (*6*). Data were right truncated to improve the fit of the detection function model. Different key functions and adjustment term combinations were considered to model the detection function (half-normal with cosine or hermite polynomial adjustment terms or either hazard rate or uniform key functions with cosine or simple polynomial adjustment terms). Various statistics (Kolmogorov-Smirnov, Cramer-von Mises, and to a lesser extent chi-squared, as it does not prioritize the fit of the detection function close to the transect line, which is the most important for unbiased estimation of detectability) and graphical diagnostics (QQ-plot and the plot of the detection function versus the actual data) were used to measure goodness-of-fit of the model. Akaike's Information Criteria (AIC) (Akaike, 1973) was used in the selection of the detection function for those models with adequate fit at distances near zero (*7*). Models were fitted separately for each survey and were stratified by the three regions within the home range (Durgun Steppe, Khuisiin Gobi and Sharga Gobi) when sample size was sufficient. In addition, to avoid positively biased

estimates of group size (that can arise if smaller groups further from the transect line tend to be missed), we fitted a regression of the natural logarithm of group size against the probability of detection at distance x from the transect line within each survey region. If the regression was statistically significant at the 15% level, then the expected group size based on the regression was used, otherwise average group size was used to estimate population density and abundance. Results included in this manuscript were obtained using the data cleaning process described above. For the purpose of a sensitivity analysis, results obtained with raw data are presented below in the Appendix Table.

3.3 Population survey results and discussion

Data was right truncated at 1 km, except for May 2017 where 5% of the data were truncated (~700 m). For the first two surveys, detection functions were fitted to stratified data. Almost all the final detection function models were half-normal with cosine adjustment terms. The exception was a uniform with cosine adjustment terms for Sharga for March 2017 and a half-normal with no adjustment terms for April 2018. With the exception of the April 2018 survey, there were indications of size bias in average group size estimation for all strata and for each of the other surveys. Thus, expected group size was used to estimate individual density and abundance for all surveys, except for April 2018. For the purpose of a sensitivity analysis, results obtained with raw data are presented below in the Appendix Table.

Appendix Table. Summary statistics of saiga distance sampling surveys conducted between January 2017 to April 2018 with the full dataset (including observations collected during deviations from the distance sampling protocol)

Date	Time (days) since first PPR confirmation	Total effort (km)	Individual (group) counts	Density of Individuals [95% CI]	Abundance [95% CI]	Expected cluster size[a] [95% CI]	Average Detection probability Live saiga[a] [95% CI]
January 2017	30	1,505	2,366 (373)	1.33 [1.01, 1.75]	28,890 [21,924–38,071]	D: 4.0 [3.3, 4.7]<br>K: 3.5 [3.0, 4.2]<br>S: 6.3 [4.8, 8.2]	D: 0.35 [0.29, 0.42]<br>K: 0.35 [0.30, 0.40]<br>S: 0.42 [0.35, 0.49]
March 2017	90	1,505	2,310 (174)	0.88 [0.54, 1.45]	19,188 [11,692–31,488]	D: 10.9 [7.9, 14.9]<br>K: 11.7 [8.7, 15.8]<br>S: 5.5 [4.3, 6.9]	D: 0.42 [0.35, 0.50]<br>K: 0.73 [0.61, 0.88]<br>S: 0.58 [0.50, 0.68]
May 2017	150	1,263	749 (159)	0.40 [0.28, 0.58]	8,772 [6,121–12,569]	D: 2.6 [2.1, 3.3]<br>K: 2.6 [2.1, 3.2]<br>S: 2.3 [1.6, 3.3]	0.51 [0.46, 0.57]
April 2018	480	1,505	369 (46)	0.24 [0.14, 0.42]	5,142 [2,929–9,028]	D: 11.7 [7.5, 18.1]<br>K: 10.9 [6.1, 19.6]<br>S: 6.3 [4.4, 8.9]	0.51 [0.40, 0.65]

b. D: Durgun Steppe, K: Khuisiin Gobi, S: Sharga Gobi

As expected, estimates were higher using the raw datasets, particularly for the January 2017 survey, however the total population decline was consistently estimated at 82%.

Despite all efforts deployed in the field and the best intentions of field personnel, only a portion of the home range of a species can be searched, and only a fraction of animals will be detected in this searched area due to the inevitable issue of imperfect detection (*7–11*). In the distance sampling analysis, the estimated detection probabilities for live animals ranged from 0.35 to 0.69. Assuming detection probabilities are similar for carcasses (although likely lower), this would suggest that the 5,425 animals collected and destroyed only represented about half of the carcasses present in the searched area, which in turn represents just a fraction of the actual outbreak area. This explains the gap observed between carcass count and total mortality estimated from population monitoring, which highlights the importance of using robust population estimation methods in the assessment of infectious disease impact (*12*).

4. References

1. Couacy-Hymann E, Roger F, Hurard C, Guillou JP, Libeau G, Diallo A. Rapid and sensitive detection of peste des petits ruminants virus by a polymerase chain reaction assay. J Virol Methods. 2002;100:17–25. PubMed https://doi.org/10.1016/S0166-0934(01)00386-X

2. Clarke B, Mahapatra M, Friedgut O, Bumbarov V, Parida S. Persistence of Lineage IV Peste-des-petits ruminants virus within Israel since 1993: An evolutionary perspective. PLoS One. 2017;12:e0177028. PubMed https://doi.org/10.1371/journal.pone.0177028

3. Baazizi R, Mahapatra M, Clarke BD, Ait-Oudhia K, Khelef D, Parida S. Peste des petits ruminants (PPR): A neglected tropical disease in Maghreb region of North Africa and its threat to Europe. PLoS One. 2017;12:e0175461. PubMed https://doi.org/10.1371/journal.pone.0175461

4. Kimura M. A simple method for estimating evolutionary rates of base substitutions through comparative studies of nucleotide sequences. J Mol Evol. 1980;16:111–20. PubMed https://doi.org/10.1007/BF01731581

5. Tamura K, Stecher G, Peterson D, Filipski A, Kumar S. MEGA6: Molecular Evolutionary Genetics Analysis version 6.0. Mol Biol Evol. 2013;30:2725–9. PubMed https://doi.org/10.1093/molbev/mst197

6. Thomas L, Buckland ST, Rexstad EA, Laake JL, Strindberg S, Hedley SL, et al. Distance software: design and analysis of distance sampling surveys for estimating population size. J Appl Ecol. 2010;47:5–14. PubMed https://doi.org/10.1111/j.1365-2664.2009.01737.x

7. Buckland ST, Anderson, D.R., Burnham, K.P., Laake, J., Borchers, D., Thomas, L. Introduction to distance sampling: estimating abundance of biological populations. Oxford (UK): Oxford University Press; 2001. p. 448.

8. Kellner KF, Swihart RK. Accounting for imperfect detection in ecology: a quantitative review. PLoS One. 2014;9:e111436. PubMed https://doi.org/10.1371/journal.pone.0111436

9. Kéry M, Schmid H. Monitoring programs need to take into account imperfect species detectability. Basic Appl Ecol. 2004;5:65–73. https://doi.org/10.1078/1439-1791-00194

10. Kéry M, Schmidt B. Imperfect detection and its consequences for monitoring for conservation. Community Ecol. 2008;9:207–16. https://doi.org/10.1556/ComEc.9.2008.2.10

11. MacKenzie DI, Kendall WL. How Should Detection Probability Be Incorporated into Estimates of Relative Abundance? Ecology. 2002;83:2387–93. https://doi.org/10.1890/0012-9658(2002)083[2387:HSDPBI]2.0.CO;2

12. McClintock BT, Nichols JD, Bailey LL, MacKenzie DI, Kendall WL, Franklin AB. Seeking a second opinion: uncertainty in disease ecology. Ecol Lett. 2010;13:659–74. PubMed https://doi.org/10.1111/j.1461-0248.2010.01472.x
